# We are ready for clinical implementation of Carbon Ion Radiotherapy in the United States

**DOI:** 10.1002/acm2.13133

**Published:** 2020-12-14

**Authors:** Chris Beltran, Richard A. Amos, Yi Rong

**Affiliations:** ^1^ Department of Radiation Oncology Mayo Clinic Jacksonville Jacksonville FL USA; ^2^ Proton and Advanced Radiotherapy Group Department of Medical Physics and Biomedical Engineering University College London London UK; ^3^ Department of Radiation Oncology Mayo Clinic Arizona Phoenix AZ USA

## 1 INTRODUCTION

In the past three decades, all carbon ion treatment centers are mainly located in Europe and Asia, yet the therapeutic benefits of carbon ion therapy were first discovered in the United States in 1970s. Clinical outcome studies are coming out in the recent years from those centers comparing carbon ion treatment with conventional photon radiotherapy or surgery.^1^, ^2^ People might have been wondering, as one article published in Wired late last year put it, “Carbon ion radiation therapy (CIRT) is being used to blast tumors all over the world. Just not in the country that invented it. Why a promising, potent cancer therapy isn’t used in the US?”^3^ The article revealed that University of Texas Southwestern Medical Center hopes to be the front‐runner of building a carbon ion treatment facility in the United States, which is estimated to cost $300 million. Just around the same time, Mayo Clinic announced its agreement with Hitachi, Ltd in building the first carbon ion treatment facility at one of Mayo’s campuses located in Jacksonville, Florida. Major stakeholders in the cancer treatment field have shown strong interests in building a CIRT facility in the United States. Our previous Parallel Opposed editorial also debated on the need of having at least one carbon ion facility in the country.^4^ Yet the question remains whether it is clinically and financially ready for construction and implementation of a CIRT facility in the United States. Herein, we have the leading figure for the Mayo Clinic CIRT project, Dr. Chris Beltran, arguing for the proposition that *CIRT is ready for clinical implementation in US*, and the world‐renowned scientist in proton therapy and ion‐beam related research, Richard Amos, arguing against the proposition.

Chris Beltran is Chair of the Division of Medical Physics; Director of the Particle Therapy Technical Operations; Professor of Medical Physics in the Mayo Clinic School of Medicine; and Consultant, Dept. of Radiation Oncology; Mayo Clinic Jacksonville FL. Prof. Beltran received his Ph.D. in Nuclear and Accelerator Physics from Indiana University in 2004. He completed his Medical Physics Residency at Mayo Clinic in Rochester MN and subsequently accepted a position at St. Jude Children’s Research Hospital. In 2011 he rejoined Mayo Clinic to lead the proton treatment planning section for their new facility. In 2020 Prof. Beltran joined the team in Mayo Clinic Florida to help lead the new particle center. In the last several years, he has focused on particle therapy, including clinical responsibilities such as commissioning the treatment planning system for the Mayo Clinic spot scanning proton facility and leading the proton treatment planning group. Prof. Beltran’s research laboratory has focused exclusively on particle therapy in the last few years with a focus on computational and translational biology.

Richard Amos is Associate Professor of Proton Therapy and Research Lead for Translational Proton Therapy Physics in the Department of Medical Physics and Biomedical Engineering, University College London, London, UK. He completed his Medical Physics Residency in London with the National Health Service (NHS) before working as a clinical radiotherapy physicist for a number of years in the United Kingdom and Canada. He also spent a couple of years involved in ion‐microbeam research at the Gray Laboratory Cancer Research Trust in the United Kingdom. In 2002 Richard joined the faculty at Loma Linda University Medical Center in California, the world’s first hospital‐based proton therapy facility, before moving to The University of Texas MD Anderson Cancer Center in 2005 to help implement and develop a proton therapy program, including the first spot scanning system in the United States. He returned to London in 2013 to take leadership roles in both the development of a national proton therapy service for the NHS and in developing translational proton therapy physics research. He is a Chartered Physicist, a Chartered Scientist, Associate Editor of the British Journal of Radiology, and a Fellow of the Institute of Physics and Engineering in Medicine.

## 2 OPENING STATEMENT

### 2.A Chris Beltran, PhD

Ion Therapy was first used at the Lawrence Berkeley National Laboratory over 50 yr ago.^5^ Unfortunately, the laboratory was closed and the United States based trials were therefore closed.^6^ Fortunately, Japan in 1994 and later Germany in 2002 opened clinical CIRT facilities to continue investigation of this promising modality. Currently there are 13 operational facilities with a few more under construction, all of which are either in Europe or Asia. Recently Mayo Clinic in Jacksonville, Florida in partnership with Hitachi announced that they would be building a proton and Carbon ion facility, the first in the United States. So yes, we are ready for clinical implementation of carbon Ion Therapy. Three critical areas to be concerned with when discussing clinical implementation are the physical dosimetric advantages of CIRT, the potential increase of the therapeutic ratio due to the radiobiological properties of CIRT, and the improved clinical outcomes that have been demonstrated with CIRT. Below is a quick summary broken down into three parts, physics and technology, radiation biology, and clinical outcomes.

#### 2.A.1 Physics and technology

The physics dose advantage of CIRT over x‐ray therapy due to the Bragg peak and is similar to the advantage of proton therapy. This is well documented,^7^ so there is not a need to spend time on this point except to say that the penumbra of CIRT is smaller than proton therapy but CIRT has a small fragmentation tail which proton therapy does not produce. The accelerator needed to produce the carbon ions is notable larger than that needed for proton therapy and hence so is the building needed to house the system. These two items increase the cost of such a facility which is often a reason given as to why such a facility should not be built. Two technological items of note is the modern computational methods such as fast Monte Carlo simulations and high computer processing speed. These advancements together lead to accurate physical dose calculation and corresponding physical parameters calculation such as linear energy transfer (LET), which facilitate incorporating advanced radiation biology models into the dose calculation and optimization procedures for CIRT.

#### 2.A.2 Radiation biology

In 2016 the National Cancer Institute held a workshop consisting of a multidisciplinary team focused on charged particle radiobiology.^8^ Although the workshop and subsequent reports had a major focus on proton therapy, a significant portion of time was spent on the radiobiological advantages of CIRT. The radiobiological advantage of CIRT comes from its high LET. While photon therapy has an LET of <1.0 keV/μm and proton ranges from 1.0 keV/μm at the entrance and up to 10 keV/μm at the Bragg peak; CIRT ranges from ~ 15 keV/μm at the entrance to >200 keV/μm at the Bragg peak. High LET of CIRT leads to direct and clustered DNA damage and hence a large relative biological effectiveness (RBE). In addition to the large RBE, which in a generic way can be between 3 and 4 near the Bragg peak, specific biological mechanisms makes CIRT especially good for tumors that are radiation resistant and/or hypoxic, and has great potential for inducing therapeutic immune responses. There is currently a lot of preclinical in‐vivo work and clinical trials being conducted to help better understand these mechanisms. This lack of a full understanding of the underlying mechanisms and their exact translation to and implication in a clinical setting is often a reason given as to why such a facility should not be built.

#### 2.A.3 Clinical outcomes

Turning to the clinical outcomes, approximately 30 000 patients have been treated with CIRT in the past two decades. Phase I/II trials have been carried out in Japan and Europe over the years and show negligible toxicity while simultaneously showing great tumor control for a wide variety of treatment sites including, but not limited to, bone and soft tissue sarcomas, adenocarcinoma, and malignant melanoma. Currently there are more than 10 ongoing clinical trials across the world, many of which are phase III trials. Recent review articles^9^, ^10^ summaries these trials, which include investigation of pancreatic, glioblastoma, lung, and many others. It has been shown that CIRT have particularly unique abilities when treating classically radiation resistant tumors. Lack of clinical evidence is often a reason given as to why such a facility should not be built.

It has been shown that we can deliver high quality and safe treatments to a group of patients with otherwise poor outcomes and few if any options. As we learn more about the biological mechanisms, these therapies can be further refined, targeted, and possibly expanded to more indications. As more centers are built, we can run more trials and show the merit of CIRT along with the limitations. Also, as more centers are built, the technology will improve and price will be reduced allowing for further expansion. Yes, there is always more to learn and the best way to do that is by expanding access which means building new centers, so yes, we are ready for FURTHER clinical implementation of CIRT.

### 2.B Richard A. Amos, MSc, FIPEM

Radiotherapy, one of the three main pillars of trimodal cancer care, is well demonstrated to improve overall survival, spare healthy organs, and improve quality of life.^11^ The last 15 yr or so has seen a proliferation of proton‐based radiotherapy centers across the United States and elsewhere purported to improve the therapeutic ratio for oncology patients. This is based on the expectation that the favorable physical dose distribution of proton therapy, compared to that photon‐based radiotherapy, allows for curative dose to tumors with reduced toxicity, or more importantly less life‐threatening toxicity. As with most new and expensive technologies, the cost of proton beam therapy (PBT) technology is reducing. Still, today there is scant randomized evidence for improved survival or quality of life in adults and should that evidence emerge, we are not yet prepared to provide PBT to a large percentage of the radiotherapy patient population nationally. Where there are data, in pediatrics, we are not yet able to provide protons to all patients who would benefit in the United States. Furthermore, should the RadComp trial examining the potential benefit of PBT for left‐sided breast cancer patients, for example, be positive, the demand would sharply outweigh the supply.^12^

So in the midst of determining the value of PBT, arguably a substantial step forward in the physical dose distribution for radiotherapy, we ask, what further benefit could we gain by expanding to CIRT? Should we abandon the financial investment and clinical trials participation so far to better understand PBT and move on, or is there a hypothesis, for a specific subgroup of patients clearly not benefitting from protons, that may be aided by CIRT? If so, is there evidence that CIRT is the best next investment, or today, is it still exciting physics and technology that is not value added in oncology?

The fundamental physical gains in using CIRT over proton beams are the greater RBE toward the end‐of‐range, the sharper Bragg peak with increased peak‐to‐plateau ratio, and tight lateral penumbrae from the reduced scatter. This means that dose to the target could potentially be escalated with far less dose to normal tissues. Where the target touches or overlaps with critical normal tissue, there may still be no significant gain. However for outcomes governed by local control, where the target is surrounded by a sea of the same normal tissue such as brain, lung, or liver, or where a high central target dose that does not touch the adjacent normal tissue may be therapeutic, CIRT could be life‐saving.

To be ready, we would need to know this hypothesis is true, know how many people are affected by this hypothesis, know whether we can provide CIRT to those with this problem, and know if the cost as a fraction of the healthcare budget considering what other public health measures might be adopted with the same investment. Data supporting the hypothesis are limited, and it has been noted that carbon ions may not be the ideal particle for this case. For example, the use of helium ions for radiotherapy is currently an active area of investigation and may prove an ideal compromise between dosimetric characteristics, clinical efficacy, and cost.^13^ There is a paucity of studies looking into how many patients are likely to benefit from the potential advantages of CIRT, although the group at the Mayo Clinic has started to address this question.^14^ Practically, getting the limited number of patients who might benefit from CIRT to a limited number of CIRT centers is another challenge, particularly in non‐nationalized healthcare systems such as that in the United States. Lastly, the cost to proliferate CIRT must consider not only the cost of the centers themselves, but also the costs already expended to proliferate PBT. Building CIRT facilities without reaching critical mass to define the need or develop expertise that might bring high risk to the center, creating pressure to fill the center with patients who would otherwise be well suited to less experimental or preliminary modalities, and stall without ever realizing the potential benefit.

So, are we ready to proliferate CIRT as a radiotherapy modality? Today, no. This endeavor needs also to address the optimal particle in a methodical and measured way, consider the investment made in PBT is seen to its meaningful conclusion treating the right patients the right way, and then move forward with solid preclinical evidence and a plan to reach the patients who benefit. Long‐term development should be based on patient needs more‐so than the whim of market pressures and scientific interest. Increased participation in international clinical trials utilizing existing facilities is prudent. I do concede that the United States should develop a national particle therapy research facility in order to contribute to international collaborative trials; to develop preclinical research programs; to investigate the merits and efficacy of various ion species, and to develop technical innovations to reduce costs associated with ion‐beam therapies.

## 3 REBUTTAL

### 3.A Chris Beltran, PhD

As I read Mr. Amos’s well written position, I came across three main points against building a carbon facility (although he does concede that the United States should develop at least one center). The first major point is that we first need more proton centers to ensure capacity meets demand. While I agree that more proton centers throughout the world would be great, I do not think this is a mutually exclusive state, in fact most, if not all, carbon centers will also be proton centers. The key indications for carbon therapy are not well treated by proton or photon therapy, so building more proton centers *first* would not address this need. The second major point is that perhaps we should first study the question whether carbon is the best ion. This question has two interesting components, first, by what criteria do we judge “better,” as there is both clinical outcomes and overall cost. For example, helium would have a lower cost, but the biological impact is smaller. Lithium is notoriously hard with injection/acceleration and heavier than carbon ions, thus creates large fragmentation. In an ideal world, all these would be clinically studied and a winner chosen, but this is impractical. For better or worse, this question was looked at when the NIRS facility was built and they settled on carbon as the best compromise. Given that we have almost 3 decades and 40 000 patients treated with carbon, I think this is the safest and most practical route. The third point is that there is not enough clinical evidence. While more is needed in terms of data quantity and variety, the only practical way to do this is to build more centers thereby increasing access, treat patients where the evidence is clear, and run trials where more evidence is required. More centers would also allow for more preclinical research by a larger pool of researchers to be conducted. As more centers are built, the clinical evidence will grow, innovation will grow, cost will shrink, and cancer patients will benefit.

### 3.B Richard A. Amos, MSc, FIPEM

Dr. Beltran has provided a concise overview of CIRT highlighting the history of the modality, the basic physical and radiobiological characteristics, and the current status of clinical trials. The physical and radiobiological characteristics of carbon ion beams do indeed suggest potential clinical benefits of CIRT compared to XRT and also PBT, however, these need to be demonstrated in clinical outcomes. While there have indeed been positive results in Phase I/II trials for a number of specific sites, there still remains a paucity of data from completed Phase III trials. Until such data indicates clear clinical benefit, I maintain that we are not yet ready for general clinical implementation of CIRT.

Dr. Beltran does elude to the high financial costs associated with the development of a CIRT facility. These costs are substantial and prohibitive to all but a few institutions, making the argument in favor of general clinical implementation of CIRT difficult from a cost‐effectiveness perspective, certainly while there remains a paucity of Phase III data. The proliferation of PBT centers in the United States has not been without its casualties with some struggling or failing financially.

There have been numerous technical developments in recent years with both XRT and PBT. Advances in image guidance have improved radiotherapy targeting accuracy, for example. Spot scanning capabilities of contemporary PBT delivery systems have enabled the development of intensity‐modulated proton therapy (IMPT) techniques, improving comformality even further. Technical developments such as these, and others, potentially improve the efficacy of existing radiotherapy modalities enabling dose escalated and/or hypo‐fractionated treatment regimens to be applied to certain indications, all at modest cost compared to CIRT. Work is ongoing to develop radiation‐drug combinations to improve clinical outcomes and reduce normal tissue toxicities.^15^ Drug development is by no means inexpensive, but once developed they can potentially reach a larger percentage of radiotherapy patients for whom these drugs are indicated. There is currently great interest in ultra‐high dose rate (FLASH) radiotherapy as a result of encouraging preclinical data.^16^ Contemporary PBT systems can be enabled to deliver FLASH dose rates; the world’s first proton FLASH trial in humans commenced in 2020 in the United States. If this and subsequent trials are positive, this also *may* lead to improved efficacy at modestly increased costs. Irrespective of these examples, for tumors potentially benefiting from the physical and radiobiological characteristics of ion‐beam therapy, can we be sure that carbon ions are the right investment?

Particle therapy has to be developed and studied thoroughly in order to reach its fullest potential and to have positive impact on outcomes for cancer patients on a public health scale. This will require the right balance between clinical efficacy and cost. Whether the optimal particle to have such a large impact is the carbon ion, the proton, or something in‐between is yet to be shown. I will watch the development and progress of CIRT at Mayo Clinic with great interest, and I commend them for being the first to develop this modality in the United States. This facility will undoubtably generate further evidence of the clinical efficacy, or lack thereof, of CIRT, and this is a valuable contribution to the field. Dr. Beltran states that they are indeed ready for clinical implementation of CIRT, but until more Phase III data are available and associated costs are reduced, I do not believe that the wider radiation oncology community is ready.
